# Sex, senescence, senolytics, and cognition

**DOI:** 10.3389/fnagi.2025.1555872

**Published:** 2025-03-04

**Authors:** Thomas C. Foster, Ashok Kumar

**Affiliations:** ^1^McKnight Brain Institute, Department of Neuroscience, University of Florida, Gainesville, FL, United States; ^2^Genetics and Genomics Graduate Program, Genetics Institute, University of Florida, Gainesville, FL, United States

**Keywords:** senolytic treatment, cellular senescence, sex differences, sex hormone, aging

## Abstract

This review focuses on sexual dimorphism in cellular senescence and senolytic treatment in relation to brain health and age-related cognitive decline. The stressors of aging, DNA damage, inflammation, and oxidative stress induce cell senescence, a hallmark of aging. Senescent cells change their function and molecular profile and are primed to release pro-inflammatory cytokines. The functional changes include the activation of cell signals to prevent cell death. The release of pro-inflammatory cytokines from peripheral senescent cells during middle age induces senescence of neighbor cells and heightens the level of systemic inflammation, contributing to neuroinflammation. In response to neuroinflammation and oxidative stress, some neurons alter their physiology, decreasing neuronal excitability and synaptic transmission. Senescent neurophysiology is protective against cell death due to excitotoxicity, at the expense of a loss of normal cell function, contributing to age-related cognitive decline. The level of peripheral cell senescence and systemic inflammation may underlie sexual dimorphism in the prevalence, symptoms, and pathogenesis of age-related diseases, including neurodegenerative diseases. Sex differences have been observed for senescence of astrocytes, microglia, and peripheral cells, including those involved in innate and adaptive immune responses. Interventions that remove senescent cells, such as senolytic drugs, can reduce or ameliorate some of the aging-related loss of function. Similarities and differences in senolytic responses of males and females depend on the system examined, the treatment regimen, the level of senescent cell burden, and the age when treatment is initiated. Estrogen impacts several of these factors and influences the transcription of genes promoting growth, proliferation, and cell survival programs in a manner opposite that of senolytic drugs. In addition, estrogen has anti-aging effects that are independent of cell senescence, including rapidly modifying senescent neurophysiology. Thus, it is important to recognize that, in addition to sex differences in cell senescence, there are other sexually dimorphic mechanisms that contribute to the aging process. The results indicate that senolytics interact with fundamental biology, including sex hormones.

## Introduction

Senescence at the cellular level plays a significant role in the decline of physical and cognitive capacities during chronological aging and the prevalence, symptoms, and pathogenesis of age-related diseases. Senolytic drugs target and remove senescent cells, and senolytic therapies have emerged as promising interventions to promote healthy aging. However, the research has described variability in the effectiveness of senolytic treatments between sexes. The emerging research highlights the importance of sex-specific approaches to studying senolytic therapies. Early findings emphasize that understanding the interplay between sex, cellular senescence, and senolytic treatment is crucial for developing personalized strategies to support cognitive health and optimize outcomes for both men and women. This review article focuses on sexual dimorphism in cellular senescence and senolytic treatment in relation to brain health and age-related cognitive decline.

## Aging systems and cellular senescence

Aging can be defined as a progressive loss of function over time, measured at the level of organs, systems, and behavior. For example, a decline in episodic memory emerges starting in middle age (Foster, 2019; Nyberg and Pudas, 2019). Animal models indicate that the decline of episodic memory is associated with a shift in hippocampal neurophysiology, altering network properties by influencing neuronal excitability and synaptic function (Foster, 2019). The shift in physiology is preceded by molecular changes associated with the stressors or hallmarks of brain aging, including a rise in inflammation and oxidative stress (Mattson and Arumugam, 2018). Other molecular changes observed in older brains may represent resiliency or homeostatic mechanisms that protect cells from the stressors of aging. These resiliency mechanisms can delay functional decline and contribute to preserved cognition (Ferrucci et al., 2018; McQuail et al., 2020; Smith et al., 2020; Yegla and Foster, 2022). Finally, some of these protective processes may preserve cell viability at the expense of a loss of function.

Cellular senescence is a state of cell cycle arrest that arises because of exhaustive cell proliferation (replicative senescence) or due to stressors, including genotoxic and oxidative damage of DNA, telomere shortening, and oncogene activation (Di Micco et al., 2021). In 1961, Leonard and Moorhead found that older fibroblast cells grown *in vitro* eventually stopped dividing (Hayflick, and Moorhead, 1961). The state of non-proliferation after extended culture was termed replication senescence. Senescence is usually described as a change in function at the cellular level in response to stressors associated with aging. The mechanisms of replication senescence involve DNA methylation or DNA damage associated with proliferation, oxidative stress, inflammation, environmental toxins, and the loss of telomeric base pairs. Importantly, the cells remained viable and metabolically active. Senescent cells undergo a transformation in which normal metabolic functions are subsumed by chronic activation of processes in response to stress, including the release of pro-inflammatory cytokines. Some of the processes initiated during senescence protect the cells through the activation of genomic programs that inhibit apoptosis (Zhu et al., 2015; Fan et al., 2020; Budamagunta et al., 2023). The shift from normal metabolism to senescence encourages the survival of cells that release pro-inflammatory factors. Thus, this adaptive stress response may preserve cell survival at the expense of an age-related loss of normal function at the macro level of the organ, system, or behavior.

The biological role of senescence is complex and depends upon physiological context. Senescence is involved in development and wound repair. In some cell types, DNA damage can transform cells to become malignant/cancerous, in which they replicate indefinitely. Cell senescence, with the inhibition of replication, represents an anti-tumor mechanism, and the pro-inflammatory stress signals attract and activate immune cells to remove senescent and cancerous cells. However, the release of pro-inflammatory factors and reactive oxygen species (ROS) can also act to induce senescence of nearby cells, a process referred to as the bystander effect (Nelson et al., 2018; Schwartz and Conboy, 2023). The number of senescent cells increases over time due to DNA damage associated with replicative senescence and exposure to external environmental genotoxins or internal stressors, such as inflammation and oxidative stress (e.g., the bystander effect). In addition, a decreased ability of the immune system to remove senescent cells contributes to an increase in senescent cell burden with chronological age.

## Markers of cellular senescence

The loss of replicative potential is generally associated with DNA methylation or damage, an increase in cell size, deposition of the lipid *β*-galactosidase (*β*-gal), and expression of cell cycle inhibitor proteins p16 and p21. In addition, senescent cells are primed to release stress signals in response to a variety of stimuli (Budamagunta et al., 2021). The collection of released molecules, pro-inflammatory cytokines and chemokines, growth factors, and matrix remodeling proteins represent the senescence-associated secretory phenotype (SASP). Senescent cells also exhibit an increase in ROS, which contributes to DNA damage and alters calcium signaling involved in cell cycle arrest and SASP production (Martin and Bernard, 2018).

Most cell types exhibit replicative senescence following multiple sequential serial passages *in vitro*. However, for *in vivo* studies, the markers of cellular senescence vary across cell types and tissues, which can create a problem for identifying senescent cells. Many non-neuronal cells exhibit a characteristic increase in expression of cell cycle inhibitor proteins p16 and p21, cytokines including IL6, IL1α, IL1β, matrix metalloproteinases, and proteins that regulate senescence and the immune response, as well as increased expression of related genes (e.g., *Cdkn1a*, *Cdkn2a*, *Il6*, *Mmp3*, and *Tnfsf11*). Some of these characteristic senescent markers are not readily apparent in the brain. *Cdkn2a* is increased in most tissues with age; however, it was undetectable in the hypothalamus of female mice (Hudgins et al., 2018). *Cdkn1a* increased in the liver and kidney but not the hypothalamus, and *Il6* expression decreased in the hypothalamus with age (Hudgins et al., 2018). *Cdkn2a* expression was observed to increase in the lung, bone marrow, kidney, and spleen, but not the dentate gyrus of the hippocampus, in aged male rats or following doxorubicin treatment of young male rats (Budamagunta et al., 2023; Budamagunta et al., 2024). Similarly, *Il6*, *Mmp3*, and *Tnfsf11* are increased in the spleen with age or following doxorubicin treatment, and under these conditions, *Il6* is not increased, and *Mmp3* and *Tnfsf11* are undetectable in the dentate gyrus (Budamagunta et al., 2023). In contrast, *Cdkn1a* is increased in the dentate gyrus of aged male rats and following doxorubicin treatment in young males and has been observed in long-term neuronal cultures (Moreno-Blas et al., 2019). It is unclear from these studies which brain cell types expressed the senescent markers. Current research is expanding the list of cell type specific senescent markers. However, due to the variability across cell types, it is recommended that researchers describe the manifestation of several markers in order to properly identify and characterize senescent cells.

Sex differences have been observed in the senescence of brain astrocytes, microglia, and peripheral cells, including those involved in innate and adaptive immune responses. Differences in senescence may account for sexual dimorphism in the propensity of some cancers and diseases, including neurodegenerative disease. Cultured astrocytes exhibit replicative senescence following serial passages, and senescence markers are induced in astrocyte cultures by oxidative stress and DNA damage (Cohen and Torres, 2019; Lye et al., 2019). Astrocyte activation in response to damage *in vivo* can result in either astrogliosis or senescence. Notably, many of the markers of astrogliosis are similar to senescence markers (Cohen and Torres, 2019). During astrogliosis, the cells release cytokines, chemokines, and growth factors. Astrocytes become hypertrophic with the growth of the cell body and processes. In contrast to senescence, astrogliosis can increase proliferation. For genotoxic damage, sex differences in the induction of replicative senescence of astrocytes influences tumorigenesis (Kfoury et al., 2018; Broestl et al., 2022). DNA damage of glioblastoma astrocytes in females results in increased expression of cell cycle inhibitor proteins, which is less likely in males. Since cell senescence is protective against tumorigenesis, sexual dimorphism in the induction of senescence likely contributes to sex differences in the incidence of cancers.

Like astrocytes, activated microglial cells exhibit several changes analogous to senescence following brain insults, infection, and aging. Microglia change shape, becoming amoeboid and releasing pro-inflammatory cytokines following activation. However, activated microglia maintain the ability to replicate, increasing in number or density, indicating that they are not senescent. With advanced age, mice exhibit an increase in microglial p16 (Ogrodnik et al., 2021), which may be greater in females (Kang et al., 2024). In addition, gene markers of neuroinflammation increase to a greater extent in the brains of older females (Berchtold et al., 2008; Mangold et al., 2017; Kang et al., 2018). Again, it is not clear that this represents senescence, as sex differences are observed in recovery from inflammation, favoring females, suggesting that the differences represent resilience mechanisms. For example, aged female mice exhibit evidence for an increase in neuroprotective microglia, and the hippocampus of aged females is more resilient to acute systemic inflammation (Barter et al., 2019; Rani et al., 2022; Polcz et al., 2023; Kang et al., 2024).

Neurons represent some of the oldest cells of the organism. These post-mitotic, non-cycling cells are less susceptible to expression of cell cycle inhibitor proteins. Long-term cultures of terminally differentiated neurons may exhibit some senescent markers such as increased *β*-gal, p16, physiological changes, and increased oxidative stress (Scott et al., 1988; Landfield, 1996; Dong et al., 2011; Ishikawa and Ishikawa, 2020). However, neurons with the “senescent phenotype” appear more resistant to oxidative stress, consistent with the idea that these modifications represent an adaptive stress response (Ishikawa and Ishikawa, 2020). *In vivo* evidence for neuronal senescence during normal aging has also been reported for Purkinje cells from very old (32 months) male mice, which exhibited a robust increase in DNA damage and the production of ROS and IL-6 (Jurk et al., 2012).

The increase in cell senescence burden and the associated increase in the release of pro-inflammatory stress signals over the course of chronological aging contribute to the chronic low-level systemic inflammation characteristic of aging. In turn, an increase in systemic inflammation is linked to functional decline in several tissues, including an increase in neuronal levels of ROS that mediate senescent neurophysiology (Bodhinathan et al., 2010a; Bodhinathan et al., 2010b; Budamagunta et al., 2023; Budamagunta et al., 2024). The increase in ROS during middle age results in constitutive or prolonged activation of redox homeostasis signaling in neurons (Kumar et al., 2018). Similar to senescent cells in the periphery (Martin and Bernard, 2018), the shift in redox state acts to shift calcium signaling in the brain (Bodhinathan et al., 2010a; Bodhinathan et al., 2010b; Kumar et al., 2019). The redox shift increases the release of calcium from internal calcium stores, which acts on potassium channels to hyperpolarize the cell, decreasing neuronal excitability. Moreover, there is a redox-mediated decrease in the entry of calcium from *N*-methyl-d-aspartate receptors (NMDARs). The resulting decrease in neuronal excitability and altered synaptic function of older animals is protective against excitotoxicity and neuronal death and is referred to as senescent neurophysiology (Foster, 2007; Kumar and Foster, 2013; Kumar et al., 2018; Foster, 2019; Chou et al., 2023). Senescent neurophysiology of CA1 neurons in the hippocampus is linked to the emergence of episodic memory deficits (Foster, 2012a,b; Foster, 2019). Thus, the neuroprotection provided by senescent neurophysiology comes at the cost of hippocampal-dependent memory function.

Sexual dimorphism is reported for the prevalence, symptoms, and pathogenesis of neurodegenerative diseases of aging. Depending on the model of neurodegenerative disease, senescent marker genes or proteins may or may not be observed in neurons (Bussian et al., 2018; Chinta et al., 2018; Musi et al., 2018; Zhang et al., 2019; Bae et al., 2022). Recent work on postmortem brains of Alzheimer’s patients suggests a novel marker, CDKN2D/p19, which is increased in neurons that express neurofibrillary tangles (Dehkordi et al., 2021). CDKN2D/p19 is thought to be involved in regulating cell growth and repressing neuronal proliferation. For studies that examine cell senescence and neurodegenerative disease of aging, it is unclear if there are sex differences in the expression of senescence markers in neurons, providing an area for further research.

## Sexual dimorphism in the response to senolytic treatment

Senolysis is the targeted removal of senescent cells, which can have beneficial or rejuvenating effects on health, possibly increasing lifespan through a decrease in age-related chronic inflammation and tissue dysfunction (Lorenzo et al., 2023). The genetic silencing of key genes that negatively regulate cell death permits apoptosis of senescent cells. The genes include ephrins (EFNB1 or 3), PI3Kδ, p21, BCL-xL, or plasminogen-activated inhibitor-2 (Zhu et al., 2015). Senolytics are therapeutic agents that selectively remove senescent cells by targeting the pro-survival pathways senescent cells use to evade apoptosis. Drugs targeting these pathways selectively kill senescent cells, but not proliferating, quiescent, or differentiated cells (Zhu et al., 2015). Navitoclax (ABT-263) and Dasatinib (D) are anti-cancer drugs. ABT-263 inhibits Bcl-2 and is effective against several senescent cell types (Chang et al., 2016). Dasatinib is an inhibitor of multiple tyrosine kinases and is particularly effective in eliminating senescent human fat progenitor cells. Quercetin (Q) is derived from plants, fruits, and vegetables and is more effective against senescent human endothelial cells and mouse mesenchymal stem cells. Fisetin, a flavonoid, is found in fruits and vegetables and inhibits the PI3K/AKT/mTOR pathway to promote apoptosis of cancer cells (Frezzato et al., 2019). Due to the importance of cell senescence in development and wound healing, chronic senolytic treatment may result in toxicity. To avoid adverse effects, drug treatments are usually cyclic on/off with 3–5 days of treatment followed by a break of 1–2 weeks. Even with cyclic treatment, the process of clearing senescent cells may lead to a temporary increase in inflammation (Garrido et al., 2022; Gonzales et al., 2023).

Similarities and differences in senolytic responses of males and females depend on the system examined, treatment regimen, differences in the level of senescent cell burden, and age when treatment is initiated. The importance of age and treatment in determining the effect of senolysis in females is illustrated by two studies that genetically removed p16 expressing cells. INK-ATTAC transgenic mice permit specific deletion of cells expressing p16. Removal of p16-expressing cells, starting in middle age, reduced the age-related increase in fat accumulation and increased spontaneous activity and exploratory behavior in both sexes (Baker et al., 2016). In contrast, mice with a genetic knockout of p16 exhibited a female-specific increase in open-field activity starting at 8 weeks postnatal (Kim et al., 2019). The researchers also found that female p16 knockout mice exhibited a decrease in weight gain, an increase in the proliferation of cerebellar neuronal cells, and an increase in the expression of estrogen receptor beta (ERβ). Since cell senescence is involved in tissue patterning during embryonic development (Rhinn et al., 2019), the results suggest crosstalk between the p16 expressing cells and estrogen receptors during early development, possibly even during maturation of adult females.

Detrimental effects have been reported, specifically for young adult females, following treatment with senolytic drugs. Repeated traumatic brain injury (TBI) increases the expression of cellular senescence markers in the brains of young adult (8–10 week old) mice (Schwab et al., 2022). A single intraperitoneal injection of ABT-263, 1-week post-injury, improved cognitive performance examine 2 weeks post-injury, and decreased expression of p21 in the brain of male TBI mice. In contrast, ABT-263 treatment increased expression of p16 in the brain for both control and TBI female mice (Schwab et al., 2022). In another example, fisetin treatment once per month from 4 to 13 months of age reduced SASP factors and improved cognition in males and had no effect in female mice (Fang et al., 2023). This same study found that a once per month treatment with D + Q, from 4 to 13 months, increased SASP expression and impaired cognition in females, with minimal effects in males.

Responsiveness may depend on liver function as well as differences in how males and females metabolize and process drugs (Miller et al., 2014). Dissimilarities in liver enzyme activity, hormone levels, and body composition can result in different levels of drug exposure. One study suggested that increasing the dose of D + Q was effective in reducing brain oxidative stress in young female mice (Faria et al., 2024). For this study, young female mice were provided cyclic (3 consecutive days every two weeks) treatment with D + Q from 1 to 14 months. This treatment paradigm likely resulted in an elevation of D + Q exposure relative to the single monthly treatment.

For older animals, similarities and differences in responsiveness depend on the system examined. Removal of p16 cells, initiated in middle age, increased lifespan, maintained the function of several organs, reduced the age-related increase in fat, and increased spontaneous activity and exploratory behavior in older animals of both sexes (Baker et al., 2016). Genetic or senolytic-induced clearance of senescent cells increases cardiovascular function during aging (Clayton et al., 2023; Mahoney et al., 2024) and improves recovery following myocardial infarction in older male and female mice (Walaszczyk et al., 2019; Salerno et al., 2022). D + Q treatment decreases senescent markers to a similar extent within the temporomandibular joint of old males and females (Zhou et al., 2021a). D + Q treatment improves the osteogenic capacity of mesenchymal stem cells from aged male and female mice (Zhou et al., 2021b).

For other systems, sexual dimorphism in senolytic effects may be linked to the level of senescent cell burden associated with aging and disease. Treatment with ABT-263 or D + Q, improved cognition in young females with gene mutations that increase senescence of astrocytes, microglia, or oligodendrocyte progenitor cells (Bussian et al., 2018; Zhang et al., 2019). Male but not female mice display long-lasting neuropathic pain behavior and an increase in senescence markers in spinal cord microglia following experimental nerve damage. The increased level of senescence was reversed by senolytic treatment in males, which relieved neuropathic pain (Muralidharan et al., 2022).

Sex differences in the level of senescent cell burden may contribute to differences in the rate of aging. Males may exhibit an increased number of senescent cells in several tissues, which may contribute to increased frequency of coronary artery disease (Yousefzadeh et al., 2020; Mury et al., 2024). Across diverse species, there are sex differences in the aging of the immune system and immune responses (Klein and Flanagan, 2016). Senescence of the immune system occurs earlier and to a greater extent in men (Marquez et al., 2020) and senescent immune cells increase the release of SASP factors (Agrawal et al., 2007). The increase in cellular senescence and the release of pro-inflammatory SASP factors may contribute to the higher level of systemic pro-inflammatory cytokines in older males (Bonafe et al., 2001; Bernardi et al., 2020; Huang et al., 2021; Parkin et al., 2023).

Adult females exhibit stronger innate and adaptive immune responses with a lower incidence of infections. In contrast, males have a higher susceptibility to acute infections and higher mortality (Gubbels Bupp, 2015; Scully et al., 2020). In a study involving aged mice exposed to infection, fisetin treatment appeared to preferentially increase survival of male mice (Camell et al., 2021). Sex differences in the peripheral immune system are thought to influence the prevalence and severity of acute and chronic inflammatory diseases that accelerate cognitive decline, including cognitive impairment following sepsis (Xu et al., 2019; Polcz et al., 2023). The possible role of cell senescence in mediating the outcome of sepsis has led to a phase 2 clinical trial to assess the efficacy of fisetin in preventing the clinical sequelae of sepsis in elderly patients (Silva et al., 2024).

Biological sex is an important factor in determining lifespan and propensity for diseases of aging. Previous work suggests that sex influences the effectiveness of treatments designed to increase longevity (Miller et al., 2014; Harrison et al., 2024). Lifespan tends to be greater for females in most animal species, and women live longer than men. D + Q and fisetin have been reported to increase lifespan in male and female mice (Xu et al., 2018; Yousefzadeh et al., 2018). However, another report indicated that fisetin failed to prolong the lifespan of mice of either sex (Harrison et al., 2024). Other factors contributing to a female advantage in longevity and differences in diseases of aging include differences in behavior (men are more likely to smoke and have car accidents as teenagers). Women commonly live longer than men; however, women experience more chronic health conditions with advancing age (Austad, 2019). Lifestyle factors linked to exercise and diet contribute to an increased likelihood of death from Alzheimer’s and heart disease.

Sex-related factors contribute to variability in cognitive aging, and sex influences the effectiveness of treatments designed to preserve cognitive function (Casaletto et al., 2020; Polcz et al., 2023). Better memory performance of females, particularly verbal memory, emerges after puberty throughout adulthood. Memory function is attenuated following menopause; however, a small female advantage is maintained from middle age to old age for humans and animal models (Yonker et al., 2003; Rentz et al., 2017; Asperholm et al., 2020; Febo et al., 2020). Longitudinal studies consistently report higher average performance for middle age or older women on tests of episodic memory, verbal recognition, and fluency tasks (McCarrey et al., 2016; Nooyens et al., 2022). Indeed, across several species, males exhibit a greater decline in episodic memory during middle age (Lundervold et al., 2014; Rentz et al., 2017; Febo et al., 2020). Cyclic D + Q or ABT-263 treatment, initiated in middle age (12 months) and continued for 6–7 months, maintained episodic memory in male rats (Budamagunta et al., 2023). In females, this same treatment did not improve cognition (Rani et al., 2024). Rather, females treated with senolytics evidenced an increase in anxiety observed as increased swim speed on the watermaze and an initial learning impairment that was ameliorated by prior training on the cue version of the watermaze. The differential effect of senolytic treatment may be linked to the decline in estradiol, which would typically decrease anxiety and protect episodic memory (Aenlle and Foster, 2010; Foster, 2012a,b; Bean et al., 2014; Renczes et al., 2020; Esperanca et al., 2024; Latorre-Leal et al., 2024).

## A link between peripheral senescence, systemic inflammation, and cognitive decline

For the studies of senolytic treatment on age-related cognitive decline, the effects of treatments on biological markers of aging were only examined in male rats (Budamagunta et al., 2023). D + Q and ABT-263 treatments protected blood–brain barrier integrity and prevented the age-related increase in microglial activation. Correspondingly, treatment was associated with a decrease in the expression of immune response and oxidative stress genes and increased the expression of synaptic genes in the hippocampus. The age-related decline in hippocampal NMDAR synaptic function (i.e., senescent neurophysiology), which underlies impaired episodic memory (Foster, 2012a,b) was rejuvenated by the senolytic treatments. The finding that senolytic treatments ameliorated senescence markers in peripheral organs and the hippocampus is consistent with the idea that the decline in NMDAR function with age is linked to peripheral cytokines, which drive neuroinflammation and oxidative stress in the brain (Kumar et al., 2018; Foster, 2019; Budamagunta et al., 2024). The role of peripheral senescence and systemic inflammation on brain health was emphasized by another study examining the effect of ABT-263 on doxorubicin-induced markers of aging and memory impairment in young male rats (Budamagunta et al., 2024). Doxorubicin exhibits limited penetration of the blood–brain barrier; rather, doxorubicin’s effects on brain function are mediated by cytokines released into the blood. These cytokines cross the blood–brain barrier to increase oxidative stress in the brain (Ren et al., 2019). As such, doxorubicin treatments promoted peripheral cell senescence and the release of pro-inflammatory cytokines but had nominal effects on brain genes that change with age. The elevation of cytokines and a corresponding increase in oxidative stress in the brain induced a redox-sensitive decrease in synaptic function and impaired memory. ABT-263 prevented the doxorubicin-induced accumulation of senescent cell markers in peripheral tissue and the rise in systemic inflammatory markers. Like doxorubicin, ABT-263 does not cross the blood–brain barrier (Yamaguchi and Perkins, 2012), and yet ABT-263 improved NMDAR synaptic function and preserved cognition following doxorubicin treatment and during aging. The results suggest that a general senolytic, acting on senescence in peripheral tissue to decrease systemic SASP factors, may be desirable over brain-specific senolytic treatment. This idea is consistent with work demonstrating that transplanting older organs induces senescence in several other organs and tissues in transplant recipients, resulting in the deterioration of multiple systems and a decline in physical and cognitive capacities (Iske et al., 2024). While senolytic treatments decrease systemic inflammation markers (Hickson et al., 2019; Budamagunta et al., 2023; Gonzales et al., 2023; Ruggiero et al., 2023), it is unknown if treatments are equally effective in reducing systemic inflammation in males and females.

Systemic inflammation plays a role in the symptoms and pathogenesis of neurodegenerative diseases, contributing to sex differences in the prevalence of these diseases (Zhu et al., 2021; Nicoletti et al., 2023). Alzheimer’s disease can be characterized into two broad types: familial early-onset Alzheimer’s disease with known genetic contributions and late-onset Alzheimer’s disease, which is influenced by multiple genetic and environmental factors. Late-onset Alzheimer’s disease is the most common form of dementia, with increased prevalence in women ≥65 years of age. In male and female patients (age ≥ 70 years) with mild Alzheimer’s disease, treatment with D + Q decreased several markers of inflammation in the blood and cerebral spinal fluid (Gonzales et al., 2023). However, the number of patients was too few to determine sex differences. Interestingly, the treatment also increased IL-6 and glial fibrillary acidic protein in the cerebral spinal fluid, possibly due to an acute inflammatory response associated with the clearance of senescent astrocytes. Moreover, patients continued to exhibit a decline in memory function, although it is unclear whether the decline was less than would have been observed in the absence of treatment. Regardless, the results raise the question of whether senolytic treatment will be of benefit once neurodegeneration is established. Similarly, it will be important to determine whether reducing systemic inflammation through senolytic treatment, starting in middle age, can delay the progression of late-onset Alzheimer’s disease.

Much of the preclinical work is directed at familial Alzheimer’s diseases with known gene mutations that induce neurodegeneration in young mice. Cyclic ABT-263 or D + Q treatments of transgenic mice expressing mutant APP, PS1, or mutant microtubule-associated protein improved cognition in young adult female mice (Bussian et al., 2018; Zhang et al., 2019; Fang et al., 2024). The recurring generation of senescent cells due to a mutation and removal by senolytic treatment could augment another mechanism of aging, stem cell depletion. Therefore, it will be interesting to determine if a treatment that is initially beneficial in young mutant mice is effective as mutant animals age.

## A role for estrogen in regulating the response to senolytic treatment

Sex differences in inflammation, the rate of cognitive decline, the prevalence of neurodegenerative disease, and cell senescence are likely linked to sex hormones. There appears to be crosstalk between senescent cells and estrogen receptors in the brain starting in early development (Kim et al., 2019), possibly even during maturation of the adult. It may be important that estrogen promotes cell growth and proliferation, and anti-apoptotic cell survival programs through the activation of signaling pathways that are inhibited by senolytic drugs. Fisetin inhibits the PI3K/Akt pathway (Frezzato et al., 2019) and estrogen’s neuroprotection in the hippocampus is mediated by increasing the activity of this pathway (Harms et al., 2001; Fan et al., 2010; Jover-Mengual et al., 2010). Genetic silencing of cell survival genes permits apoptosis of senescent cells and estrogen increases expression of cell survival genes including Bcl-2 (Stoltzner et al., 2001; Zhao et al., 2004; Foster, 2005; Aenlle et al., 2009; Bean et al., 2014; Zhu et al., 2015). This crosstalk between estrogen and senolytic drugs may contribute to the detrimental effect of treatment observed in young adult females.

In adult females, sex hormones are protective against cell senescence, and a rise in SASP factor expression occurs after menopause (Ng and Hazrati, 2022). Preserved cognition in middle-aged females likely involves estrogen since impairment in episodic memory is increased after menopause in women (Jacobs et al., 2016; Rentz et al., 2017) or following removal of the ovaries in middle age rodents (Bean et al., 2015). Interestingly, senolytic treatment may hasten ovarian aging and the loss of follicles, the primary source of estrogen (Hall, 2015; Xia et al., 2024) suggesting that senolytic treatment may be contraindicated in middle-aged females.

The loss of estrogen in older females corresponds to an increase in the expression of senescence markers, as well as heightened levels of chronic inflammatory diseases and oxidative stress. Furthermore, as estrogen declines, the disparity in the expression of senescence markers between males and females becomes less pronounced. Moreover, estrogen-induced signaling and gene regulation change over the course of aging due to a shift in expression of the various estrogen receptors GPER1, ERα, and ERβ (Foster, 2012a,b; Bean et al., 2014; Kumar and Foster, 2020) and epigenetic modifications associated with long-term estrogen deprivation (Ianov et al., 2017; Barter and Foster, 2018; Sinha et al., 2021). The shift in signaling with advanced age may underlie a decrease or reversal of estrogen’s protective effects. For example, in cultures of vascular smooth muscle cells, estrogen inhibited senescence induced by oxidative stress in young females (2 months) and had senescent-promoting action in females of advanced age (18 months) (Zhu et al., 2011).

**Figure 1:**
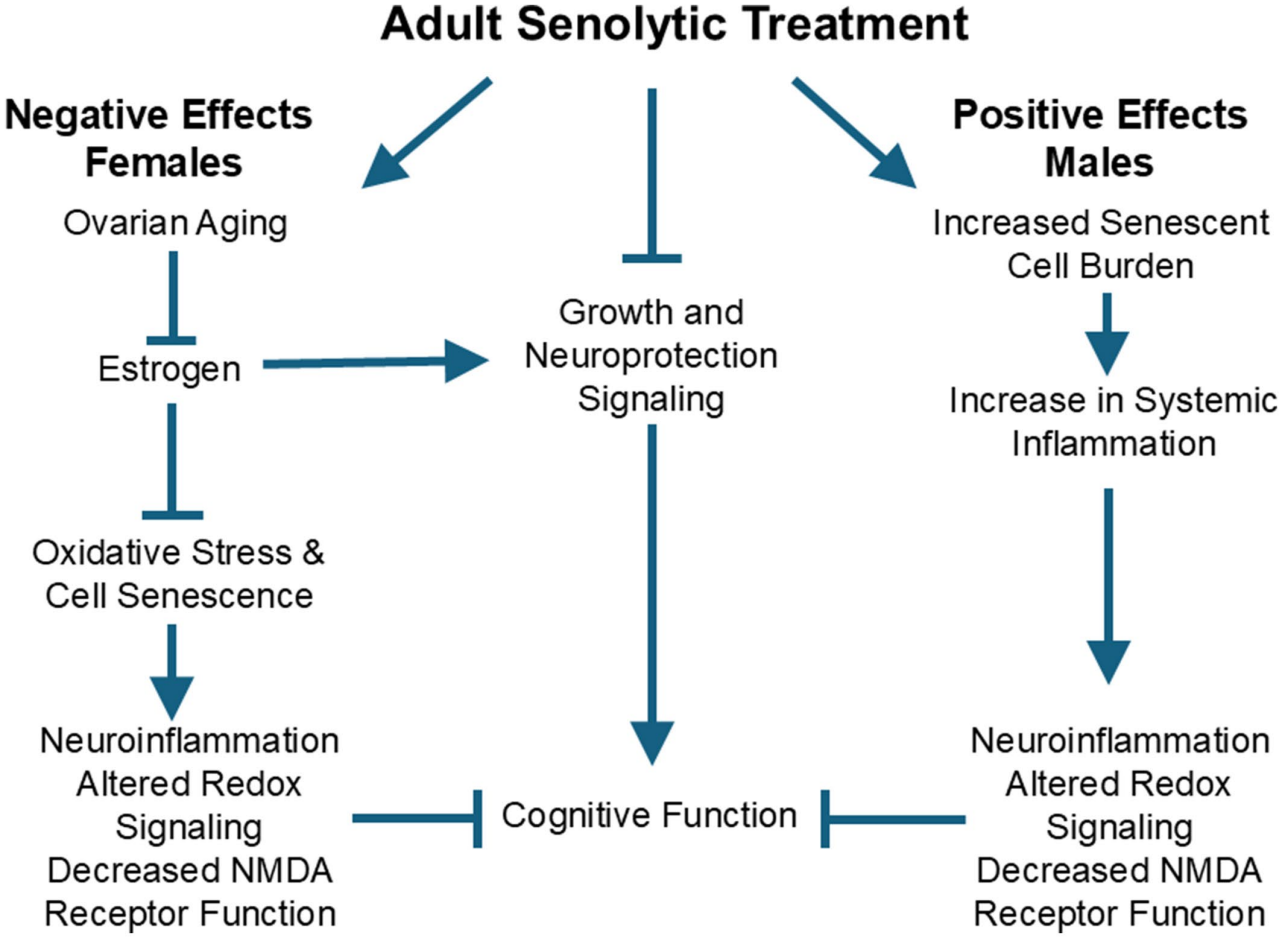
Sexual dimorphism is observed in the effectiveness of adult senolytic treatment on subsequent age-related cognitive decline. The male advantage for senolytic treatment may be due to increased levels of senescent cells and associated systemic inflammation, inducing senescent neurophysiology involved in cognitive decline. Females may be disadvantaged by treatment due to senolytic-mediated acceleration of ovarian aging and the loss of estrogen. Estrogen is protective against cognitive aging in women and guards against oxidative stress and the induction of cell senescence. Moreover, estrogen promotes cell growth and neuroprotection through signaling pathways, which are inhibited by senolytic treatment.

Together, evidence indicates that males receive a preventative benefit from early senolytic treatment. In females, early senolytic treatment may exhibit counterproductive crosstalk with estrogen signaling and may accelerate ovarian aging and the decline in estrogen and its protective effects (Figure 1). With advanced age, the loss or shift in estrogen signaling and increase in senescence markers suggests that senolytic treatment could be therapeutic when delivered to older females. This idea remains to be tested.

Finally, sexual dimorphism is observed for several biomarkers of aging, proteostasis, mitochondrial dysfunction, oxidative stress, epigenetic and immunological markers, and behavioral measures of frailty, which may be independent of cell senescence or may precede or follow induction of senescence (Horvath et al., 2016; Gordon et al., 2017; Jenkins et al., 2020; Tower et al., 2020; Hagg and Jylhava, 2021; Phyo et al., 2024). In this regard, it is important to recognize that sex hormones maintain youthful physiology and function through other mechanisms beyond cell senescence, promoting growth and reducing inflammation and oxidative stress. For example, estrogen has rapid effects on neurophysiology, opposite that observed during aging, increasing neuronal excitability, increasing the NMDAR component of synaptic transmission, and promoting long-term synaptic potentiation over long-term synaptic depression (Kumar and Foster, 2002; Foster, 2012a,b; Bean et al., 2014; Bean et al., 2015; Kumar et al., 2015). Thus, age-related changes in function due to the loss of estrogen may contribute to senescence; however, the loss of estrogen can also influence biological markers of aging that are independent of cellular senescence. This idea is evident in a study demonstrating that the loss of estrogen and increase in cellular senescence independently regulate the pathogenesis of osteoporosis (Farr et al., 2019). The field is still evolving, and more targeted studies are needed to fully understand how estrogen can regulate the response to senolytic treatments and how it might be leveraged for more effective therapies in aging populations.

Targeting senescent cells to alleviate age-related diseases and improve overall health represents a promising new therapeutic approach. However, research increasingly underscores the significance of sex in determining the efficacy and mechanisms of treatment. Estrogen, acting on metabolism, the level of senescent cell burden, and associated immune responsiveness, is a likely contributor to sexual dimorphism in the prevalence, symptoms, and pathogenesis of age-related diseases, including neurodegenerative diseases. The possible convergence of estrogen signaling with mechanisms for senescence and senolytic treatment highlights the need to explore further the influence of sex hormones and senolytic treatment in research on aging. While senolytic therapy holds transformative potential, further investigation into sex-specific responses is essential to unlocking their full benefits and advancing equitable, personalized treatments for aging and its associated diseases.
